# Reduced rhinovirus-specific antibodies are associated with acute exacerbations of chronic obstructive pulmonary disease requiring hospitalisation

**DOI:** 10.1186/1471-2466-12-37

**Published:** 2012-07-31

**Authors:** Stephanie T Yerkovich, Belinda J Hales, Melanie L Carroll, Julie G Burel, Michelle A Towers, Daniel J Smith, Wayne R Thomas, John W Upham

**Affiliations:** 1 School of Medicine, The University of Queensland, Brisbane, Australia; 2 Queensland Lung Transplant Service, The Prince Charles Hospital, Brisbane, Australia; 3 Telethon Institute for Child Health Research, Centre for Child Health Research, University of Western Australia, Perth, Australia; 4 Department of Respiratory Medicine, Princess Alexandra Hospital, Brisbane, Australia; 5 The University of Queensland School of Medicine, Princess Alexandra Hospital, Ipswich Road, Woolloongabba, Brisbane, Qld 4102, Australia

## Abstract

**Background:**

Acute exacerbations of chronic obstructive pulmonary disease (AECOPD) are often linked to respiratory infections. However, it is unknown if COPD patients who experience frequent exacerbations have impaired humoral immunity. The aim of this study was to determine if antibodies specific for common respiratory pathogens are associated with AECOPD.

**Methods:**

Plasma was obtained from COPD patients when clinically stable. AECOPD requiring hospitalisation were recorded. IgG_1_ antibodies to *H. Influenzae* outer membrane protein 6 (P6), pneumococcal surface protein C (PspC) and the VP1 viral capsid protein of rhinovirus were measured.

**Results:**

COPD patients who had an AECOPD (n = 32) had significantly lower anti-VP1 IgG_1_ antibody levels when stable compared to COPD patients who did not have an AECOPD (n = 28, p = 0.024). Furthermore, the number of hospitalisations was inversely proportional to anti-VP1 antibody levels (r = −0.331, p = 0.011). In contrast, antibodies specific for P6 and PspC were present at similar concentrations between groups. Plasma IL-21, a cytokine important for B-cell development and antibody synthesis, was also lower in COPD patients who had an AECOPD, than in stable COPD patients (p = 0.046).

**Conclusion:**

Deficient humoral immunity specific for rhinoviruses is associated with AECOPD requiring hospitalisation, and may partly explain why some COPD patients have an increased exacerbation risk following respiratory viral infections.

## Background

Acute exacerbations of chronic obstructive pulmonary disease (COPD) are responsible for much of the morbidity, mortality and health care costs associated with COPD. Exacerbations are associated with poor clinical outcomes including accelerated decline of lung function [1], reduced quality of life [2] and an increased risk of death [3]. Despite the clinical importance of exacerbations, it is not entirely clear why some COPD patients experience frequent exacerbations, while others remain relatively stable. Though exacerbations tend to become more frequent in those with poor lung function, it has recently been shown that the single best predictor of exacerbations is a history of previous exacerbations [4]. Susceptibility to exacerbations is also associated with bacterial colonisation of the airways during periods of clinical stability [5], with the presence of gastro-oesophageal reflux and with an elevated white blood cell count [4].

Many COPD exacerbations are triggered by respiratory infections with bacteria such as *Haemophilus influenzae* and *Streptococcus pneumoniae* frequently cultured from sputum [5]. In addition, the development of sensitive molecular detection methods has led to an increasing appreciation of the importance of respiratory viruses as triggers of exacerbations; human rhinoviruses are the most common viruses identified in this situation [6,7].

Some patients with COPD appear unusually susceptible to microbial pathogens, though the mechanisms mediating this susceptibility are not well understood. Hence there is a need for a more detailed analysis of anti-microbial immunity in COPD, and the extent to which this is associated with exacerbations. We hypothesized that those COPD patients with a relative baseline deficiency in circulating antibodies specific for common viral and bacterial pathogens would be at greater risk for COPD exacerbations.

Therefore, the aim of this study was to measure the concentrations of IgG_1_ antibodies specific for conserved antigens within human rhinoviruses, *H. influenzae* and *S. pneumoniae* in a group of COPD patients studied at a time of clinical stability, and to relate this to the presence or absence of exacerbations requiring hospitalisation over a twelve month period. This is pertinent as COPD patients who are hospitalised with an exacerbation have a higher mortality rate over subsequent years compared to COPD patients not hospitalised [8]. The study focused on antibodies specific for the following immunogenic proteins: (i) outer membrane protein 6 (P6) of *H. influenzae,* because reduced concentrations of anti-P6 IgG_1_ antibody are a risk factor for asthma exacerbations in children [9], (ii) pneumococcal surface protein C (PspC), because anti-PspC antibodies can mediate host protection against *S. pneumoniae*[10], and (iii) a type A conserved rhinovirus outer capsid protein (VP1) as type A rhinoviruses are the most common, and anti-VP1 antibodies exhibit cross-neutralizing activity across different rhinovirus strains *in vivo*[11]. Furthermore, because IL-21 is a cytokine that is important for B-cell development and antibody synthesis [12], circulating IL-21 was also measured.

## Methods

### Patient recruitment

We recruited sixty COPD patients as previously described [13], all of whom met ERS/ATS criteria for a clinical definition of COPD. Patients with other lung diseases or malignancy were not enrolled. The severity of COPD was graded as per the global initiative for chronic obstructive lung disease (GOLD) criteria: all patients had GOLD stage 2, 3 or 4 disease [14]. Exacerbations were defined by standard criteria as two out of three of increased sputum production, increased dyspnoea or change in sputum colour [14]. Full details of the cohort have been described previously [13]. This study was approved by the Human Research Ethics Committee, Princess Alexandra Hospital, Australia and patients provided informed written consent prior to their enrolment in the study.

### Blood sampling

Blood samples were collected from patients at a time when they had been clinically stable for at least 6 weeks. Importantly, blood sampling was not performed within 6 weeks of finishing a course of oral steroids. Plasma was stored at −20°C for later batch analysis.

### Antigen preparation

The P6 outer membrane protein of *H. influenzae* from the Eagen isolate and VP1 from human rhinovirus 1B (rhinovirus species A) were produced as fusion polypeptides with N-terminal hexa-histidine tags in pQE-80 L (Novagen, Madison, USA). PspC was derived from the pneumococcal D39 strain (aa 1–445) and cloned with a C-terminal six-histidine tag in pET20b (Novagen). The pQE-80 L and pET20b-based constructs were expressed in BL21 Star (DE3) pLysS (Novagen) using 1 mM isopropyl-b-D-thiogalactopyranoside (IPTG), in the presence of 100 μg/ml ampicillin and 34 μg/ml chloramphenicol (Invitrogen Corp., Carlsbad, USA). The expressed recombinant proteins were purified under non-denaturing conditions using Ni^2+^-nitrilotriacetic acid (Ni-NTA) agarose chromatography (Qiagen GmbH, Germany), according to the manufacturer’s protocols. Fractions containing the relevant protein were pooled and further purified using anion/cation and size exclusion chromatography. The purities of all the proteins were checked on a 12.5% sodium dodecyl sulfate-polyacrylamide gel and the concentrations determined using the optical density at 280 nm (OD280) measurements and extinction coefficients.

### Measurement of specific antibodies

Anti-P6 IgG_1_ antibodies, anti-PspC IgG_1_ antibodies and anti-VP1 IgG_1_ antibodies were measured using dissociated-enhanced immunofluoresence assay (DELFIA™) as described previously [15]. The limit of detection was 100 ng/ml. Patients who had values below the limit of detection were assigned a value of half the lower limit of detection [16].

### Measurement of CRP and IL-21 levels

CRP was measured as an index of systemic inflammation by the hospital pathology service using commercial auto-analysers. IL-21 was measured in plasma using a commercial ELISA kit (eBiosciences, San Diego, CA) according to the manufacturer’s instructions. The lower detection limit of this assay was 15.5 pg/ml.

### Statistical analysis

Data was assessed using Stata v11 (StataCorp, USA) with p < 0.05 considered statistically significant. As the data was not normally distributed, group differences were assessed using Kruskal-Wallis test, Mann–Whitney U test for unpaired responses or Fisher’s Exact Test, as appropriate. Correlations between variables were assessed using Spearman’s rank test. Simple and multivariate linear regressions were performed with exacerbation history as the dependent variable. Variables where the residuals were not normally distributed were natural log transformed. Simple linear regression analysis was initially used to evaluate the relationship between variables and exacerbation history and those variables in which p < 0.1 were then subjected to multiple linear regression analysis. The final model was obtained by backwards selection, retaining predictors that were statistically significant at α = 0.05.

## Results

### Subject demographics

The demographic characteristics of the patients are shown in Table 1. The patients were middle aged to elderly (median age 69 years). All had smoked, though less than 30% were currently smoking. Thirty-two COPD patients had at least one exacerbation requiring hospitalisation during the 12 month study period (median = 2; interquartile range 1 – 4.5) and are hereafter referred to as ‘exacerbation-prone COPD’. The majority of exacerbations occurred in winter and spring, times when multiple respiratory pathogens are circulating in the community. Twenty-eight COPD patients were free of exacerbations requiring hospitalisation during this period, and are referred to as ‘stable COPD’. Importantly, the exacerbation-prone and stable COPD patients did not differ significantly in age, sex, pack years of smoking or inhaled steroid usage, though the exacerbation-prone COPD patients had more severe airflow limitation than the stable COPD patients (p = 0.001).

**Table 1:** Subject Characteristics

	**Stable COPD Patients n = 28**	**Exacerbation-prone COPD Patients n = 32**	**p value**
Age,	69.8	69.6	0.917
median (IQ range)	(62.9 – 73.4)	(62.8 – 74.2)	
Male, n (%)	15 (54)	21 (66)	0.431
Smoker current, n (%)	8 (26)	9 (28)	1.00
Smoking pack years, median (IQ range)	53 (37–72)	45 (35 – 72)	0.721
FEV1 % predicted, median (IQ range)	46 (35 – 60)	31 (21 – 45)	0.001
Inhaled steroids, n (%)	19 (68)	26 (81)	0.251
Number of hospitalisations, median (IQ range)	0	2 (1 – 4.5)	

### Rhinovirus-specific antibody concentrations

**Figure 1:**
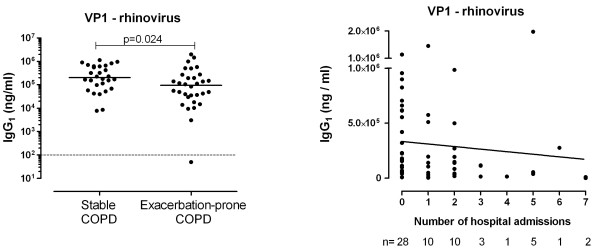
** Anti-rhinovirus IgG**_**1**_**antibodies in COPD.** (**A**) IgG_1_ antibody levels specific to rhinovirus (VP1) are plotted for stable and exacerbation-prone COPD patients with the medians indicated. The lower limit of detection is highlighted with a dotted line. Significance was assessed by Mann–Whitney test with significant differences indicated. (**B**) The relationship between the number of hospitalisations and IgG_1_ antibody levels specific to rhinovirus (VP1) is shown with the regression line. The correlation was assessed using the Spearman rank test.

IgG_1_ antibodies specific for the VP1 rhinovirus capsid antigen could be detected in the plasma of almost all the study participants (Figure 1A). Exacerbation-prone COPD patients had significantly lower concentrations of anti-VP1 IgG_1_ antibody than stable COPD patients (p = 0.024). When the anti-VP1 IgG_1_ antibody concentrations were divided into tertiles, those in the lowest tertile had the highest risk of hospitalisation over 12 months (median = 2 admissions), while those in the middle tertile (median = 0 admissions) and upper tertiles (median = 0 admissions) had significantly fewer hospitalisations (lower tertile vs middle tertile, p = 0.02; lower tertile vs upper tertile, p = 0.02; middle tertile vs upper tertile, p = 0.8). Similarly, the number of hospitalisations was inversely proportional to the anti-VP1 IgG_1_ antibody concentration (r = −0.331, p = 0.011; Figure 1b). It is noteworthy that the only patient to have no detectable anti-VP1 IgG_1_ antibodies had the highest number of hospital admissions (n = 7).

### Haemophilus- and pneumococcus-specific antibody concentrations

**Figure 2:**
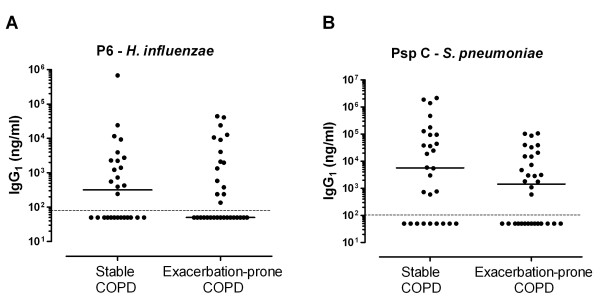
**IgG**_**1**_**antibodies to common bacterial respiratory pathogens in COPD IgG**_**1**_**antibody levels to (A)*H. influenzae*(P6) and (B) pneumococcal surface antigen (PspC) are plotted for stable and exacerbation-prone COPD patients, with the medians indicated.** The lower limit of detection is highlighted with a dotted line

IgG_1_ antibodies specific for the *H. influenzae* antigen P6 could be detected in plasma in 53% (15/28) of stable and 47% (15/32) of exacerbation-prone individuals with COPD (Figure 2). PspC-specific IgG1 antibodies could be detected in 68% (19/28) of subjects with stable COPD and 56% (18/32) of COPD subjects prone to exacerbation. Plasma concentrations of P6 and PspC antibodies tended to be slightly lower in exacerbation-prone COPD patients than in stable COPD patients, but these differences were not statistically significant (Figure 2).

### Associations between anti-VP1 IgG_1_ and clinical variables

There was a modest inverse relationship between anti-VP1 IgG_1_ and age (r = −0.281, p = 0.030), such that older individuals generally had lower anti-VP1 IgG_1_ than younger individuals (Table 2). However it should be emphasised that there was no difference in age between the exacerbation-prone and stable COPD patients. Anti-VP1 IgG_1_ antibody concentrations did not vary significantly in relation to FEV_1_, sex, current or previous smoking history, season of blood sampling or CRP (Table 2).

**Table 2:** Association between anti-VP1 antibodies and clinical factors

	***Correlation with anti-VP1 antibodies***
Age (years)	**r = −0.281, p = 0.030**
FEV1 % predicted	r = 0.224, p = 0.085
CRP (mg/L)	r = −0.159, p = 0.239
Smoking history (pack years)	r = 0.171, p = 0.196
*Comparison of anti-VP1 antibodies between groups*	*p value*
Sex (male vs female)	p = 0.774
Season of blood sampling (summer, winter, spring, autumn)	p = 0.351

In order to understand factors that predict exacerbation history, univariate and multivariate linear modelling was performed and is summarised in Table 3. Both low lung function and low anti-VP1 IgG_1_ levels were independent predictors of more frequent exacerbations.

**Table 3:** Factors associated with exacerbation frequency

***Univariate analysis***	***β***	***p***	***95% CI for β***
*FEV1 % predicted	−1.664	0.002	−2.700 – −0.628
*anti-VP1 IgG_1_ levels	−0.440	0.001	−0.700 – −0.179
Use of inhaled steroids	0.978	0.092	−0.164 – 2.119
***Multivariate analysis***	***β***	***p***	***95% CI for β***
*FEV1 % predicted	−1.388	0.007	−2.378 – −0.398
*anti-VP1 IgG_1_ levels	−0.374	0.004	−0.625 – −0.123

* variables were natural log-transformed.

Only variables with p < 0.01 are shown in the table. r^2^ of the multivariate linear regression = 0.266.

### Circulating IL-21 is significantly lower in exacerbation-prone COPD patients

**Figure 3:**
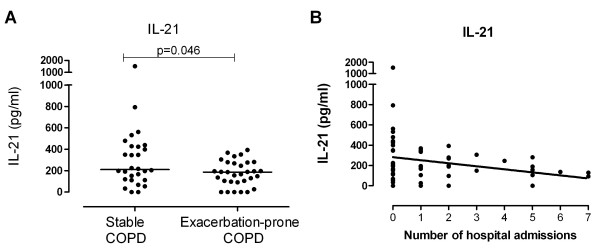
**Circulating IL-21 is lower in exacerbation-prone COPD patients Plasma IL-21 levels are plotted for (A) stable and exacerbation-prone COPD patients with the median indicated and (B) against the number of hospital admissions.** Significant differences are indicated.

In order to assess potential mechanisms responsible for the reduced rhinovirus-specific antibody levels observed in exacerbation-prone COPD patients, we measured IL-21 in plasma. Exacerbation-prone COPD patients had significantly lower IL-21 than stable COPD patients (p = 0.046, Figure 3A). Furthermore, plasma IL-21 levels were inversely proportional to the number of AECOPD requiring hospital admission (r = −0.279, p = 0.032, Figure 3B). While IL-21 was negatively correlated to smoking pack year history (r = −0.286, p = 0.030), IL-21 levels did not vary in relation to age, sex, FEV_1_, CRP or current smoking status (data not shown).

## Discussion

The key finding to emerge from this study was that exacerbation-prone COPD patients have significantly lower circulating anti-VP1 IgG_1_ and significantly lower plasma IL-21 than stable COPD patients. Interestingly exacerbation-prone and stable COPD patients had similar concentrations of IgG_1_ antibodies specific for the two bacterial antigens examined in this study, so there was no evidence of a generalised deficiency of humoral immunity to other common respiratory pathogens.

Previous studies in healthy individuals have shown that circulating rhinovirus-specific antibodies prior to virus exposure are related to subsequent symptom severity and duration during an infection [17], highlighting the importance of humoral immunity against rhinoviruses. Moreover, it has recently been shown in an animal model that anti-VP1 antibodies exhibit cross-neutralizing activity across different rhinovirus strains [11]. We therefore propose that the lower concentrations of anti-VP1 IgG_1_ observed in exacerbation-prone COPD patients places them at greater risk of infections due to a variety of rhinovirus strains, and that this may contribute to increased risk for COPD exacerbations. There is strong evidence that COPD exacerbations in which a virus is identified are more likely to result in hospital admission [6], and that rhinoviruses are the most frequent virus detected in this situation [7]. Rhinovirus infections may also induce more symptoms and a greater inflammatory response in COPD patients than in healthy individuals [18].

We considered whether other variables might have confounded our results, but found no evidence that sex, smoking history or season was related to anti-VP1 IgG_1_ antibody concentrations. While increasing age was associated with lower anti-VP1 IgG_1_, it should be emphasised that the exacerbation-prone and stable COPD patients were of similar age and the significant difference in VP1 antibodies levels between the two groups remained when corrected for age. Though exacerbation-prone COPD patients had more severe airflow obstruction than the stable COPD patients, the association between lower anti-VP1 IgG_1_ antibody concentrations and COPD exacerbations appeared to be independent of FEV_1_ (Table 2 and 3).

In seeking to understand the mechanisms that might lead to lower concentrations of anti-VP1 IgG_1_ antibody we also measured circulating IL-21. This cytokine is thought to be important for B-cell development and antibody synthesis [12]. Exacerbation-prone COPD patients certainly had significantly lower plasma IL-21 than stable COPD patients, but there was no statistically significant association between IL-21 and anti-VP1 IgG_1_ antibody concentrations in our patients. This might be explained by a lack of statistical power, or alternatively, deficient synthesis of IL-21 might be linked to COPD exacerbations via other mechanisms. There is evidence that IL-21 is also critical for CD8 T-cell memory [19], so future studies will need to assess the relationship between IL-21, CD8 T cell function and COPD exacerbations.

We can speculate that inflammatory cytokines and oxidative stress might interfere with antibody synthesis, and this is an issue that warrants further study. Systemic inflammation (as measured by CRP) was not related to anti-VP1 IgG_1_ antibody concentrations. Recent reports indicates that airway epithelial cells from patients with COPD are more susceptible to rhinovirus infection *in vitro*[20], and that experimental rhinovirus infection induces less innate interferon-α/β/λ synthesis in lung lavage cells from subjects with COPD, as compared to healthy subjects [18]. However, little attention to date has been given to adaptive immunity to rhinoviruses and how this might impact on the risk of COPD exacerbations.

One limitation of our study is that we do not have information on whether the exacerbation-prone COPD patients might have isolated themselves from contact with children, and therefore had less exposure to rhinoviruses. While social isolation might theoretically explain lower anti-VP1 IgG1, one would expect that isolation would also lead to lower exposure to *H. influenzae* and *S.pneumoniae* and thus lower detection of anti-PspC and anti-P6 IgG_1_ antibodies, but this was not observed. Secondly, the cross-sectional nature of this study does not allow the antibody stability to be determined, and further longitudinal studies will be required to address this. Finally, exacerbations requiring hospitalisation was used to determine frequent exacerbators. While this selects a subset of severe exacerbations, we do not know whether low anti-VP1 antibodies are associated with milder exacerbations that resolve without requiring hospital admission.

## Conclusion

In conclusion, because rhinoviruses are so common, it is easy to see how an impaired capacity to generate antibodies directed to a protein that is cross-neutralising across multiple rhinovirus strains could have an adverse impact on the risk of COPD exacerbations. The mechanism leading to such a specific deficit in humoral immunity to rhinoviruses warrants further investigation.

## Competing interests

The authors declare they have no competing interests.

## Authors’ contributions

STY carried out the study and performed the statistical analysis. BJH designed the DELFIA assays and carried out the study. MLC carried out and analysed the laboratory component of the study. JGB carried out and analysed the laboratory component of the study. MAT recruited patients, collected samples and compiled patient information. DJS recruited patients and compiled patient information. WRT also designed the DELFIA assays and provided intellectual input in relation to data interpretation. JWU was responsible for the study and provided intellectual input in relation to data interpretation. All authors assisted with writing the manuscript, and read and approved the final manuscript.

## Pre-publication history

The pre-publication history for this paper can be accessed here:

http://www.biomedcentral.com/1471-2466/12/37/prepub
